# Isolation Defines Identity: Functional Consequences of Extracellular Vesicle Purification Strategies

**DOI:** 10.1002/adhm.202504684

**Published:** 2026-01-19

**Authors:** Christian Preußer, Dolores J. Salander, Witold Szymanski, Johannes Graumann, Yukai Wang, Kai Zhao, Jörg W. Bartsch, María Gómez‐Serrano, Daniel Bachurski, Silke Reinartz, Elke von Pogge von Strandmann

**Affiliations:** ^1^ EV‐iTEC Core Facility Center for Tumor Biology and Immunology Marburg University Marburg Germany; ^2^ Institute for Tumor Immunology Center for Tumor Biology and Immunology Marburg University Marburg Germany; ^3^ Institute of Translational Proteomics & Core Facility Translational Proteomics Marburg University Marburg Germany; ^4^ Department of Neurosurgery Marburg University Marburg Germany; ^5^ Department I of Internal Medicine Center for Integrated Oncology Aachen Bonn Cologne Duesseldorf CECAD Center of Excellence on Cellular Stress Responses in Aging‐Associated Diseases Faculty of Medicine and University Hospital Cologne University of Cologne Cologne Germany; ^6^ Translational Oncology Group Center for Tumor Biology and Immunology Marburg University Marburg Germany

**Keywords:** ADAM10 activity, extracellular vesicles, MISEV2023, proteome, standardization

## Abstract

The biological activity of extracellular vesicles (EVs) is largely defined by their molecular cargo, yet the impact of isolation workflows on EV proteomes and function remains incompletely understood. Here, we compared four isolation strategies for EVs derived from malignant ascites and ES‐2 ovarian cancer cell culture supernatants, assessing yield, particle size, protein cargo, and EV‐associated enzymatic activity. Proteomic analyses of particle‐normalized preparations were performed according to MISEV2023 guidelines, and vesicle‐associated protease activity was profiled using a FRET‐based assay with inhibitor panels. Principal component and overlap analyses identified a common EV proteome signature for ascites and ES‐2 EVs, which was complemented by workflow‐dependent detection of additional proteins. Ultracentrifugation/density gradient (UC‐DG) and tangential flow filtration/size exclusion chromatography (TFF‐SEC) achieved the highest enrichment of canonical EV markers, whereas TFF/ultrafiltration (TFF‐UF) was enriched in lipoproteins and secreted proteins. Functionally, UC‐DG and TFF‐SEC samples exhibited strong ADAM10‐associated activity, while TFF‐UF retained residual non‐metalloprotease activity. These results reveal to what extent EV purification methods impact both, EV composition and function. This methodological awareness is critical for advancing EV‐based biomarker discovery, diagnostics, and therapeutic platforms.

## Graphical abstract

Four extracellular vesicle purification strategies are compared using ovarian‐cancer ascites and ES‐2 cell supernatants. A novel workflow links purification to function by combining particle‐normalized proteomics with matched cell‐free and cell‐based assays. A shared EV core proteome is observed, while markers, contaminants, and ADAM10 activity shift by method. This study supports evidence‐based selection for EV‐based biomarker discovery, diagnostics, and therapeutic platforms.

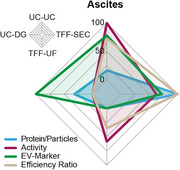

## 1 Introduction

The technological arsenal used for extracellular vesicle (EV) isolation has evolved rapidly over the past decade, with an increasing number of approaches aiming to separate EVs from complex biological matrices and to distinguish between EV subpopulations. While this methodological expansion has led to important technical and conceptual advances, it has also introduced new challenges, most notably the need to strike a balance between yield, purity and EV function. The majority of isolation techniques still require users to prioritize either high particle recovery at the cost of specificity, or high‐purity preparations with substantially lower particle counts, which come with limitations in subsequent analyses. This compromise has direct consequences for the reproducibility and translational potential of healthcare materials, including diagnostics, drug delivery, and regenerative medicine.

Arguably, differential ultracentrifugation (UC) remains the most widely used method, which, despite its extensive utilization and scalability, subjects vesicles to shear forces with the potential to compromise membrane integrity [1, 2, 3]. In addition, UC offers limited specificity, particularly when employed on biofluids comprising high concentrations of extracellular proteins and non‐EV nanoparticles such as lipoproteins [4]. Density gradient (DG) ultracentrifugation (e.g., sucrose or iodixanol) is an established method of achieving separation by exploiting differences in density, but it is considerably more laborious and time‐consuming. In reaction to these limitations, alternative workflows, such as tangential flow filtration (TFF), size exclusion chromatography (SEC), and various combinations have been introduced, aiming at gentler processing conditions and improved scalability, but also co‐isolation of non‐vesicular components. Nevertheless, each approach can introduce method‐specific biases in EV preparations (e.g., shear‐ or membrane‐dependent losses with TFF; lipoprotein co‐elution and column‐dependent recovery with SEC) [5, 6, 7]. Such method‐specific biases not only affect analytical results but also directly impact the suitability of EV preparations for biomedical applications.

Selection of an EV isolation strategy has consequences that extend beyond considerations of particle count or size distribution. It is becoming increasingly evident that isolation methodologies also exert an influence on the proteomic composition, structural integrity, and biological function of EVs [8, 9, 10]. Effects include the activity of membrane‐bound proteins such as metalloproteinases, which may serve as both biomarkers and mediators of EV function. Furthermore, recent studies have highlighted the prevalence of non‐vesicular proteins that associate with the surface of EVs, forming what is often referred to as the “EV corona”. In line with recent studies demonstrating functional protein coronas on extracellular vesicles, this corona is indicative of a biologically relevant interface between EVs and their environment with the potential to mediate interactions with recipient cells or to modulate vesicle stability and function [11, 12, 13, 14]. Rather than being considered contamination, the corona may thus represent a functional determinant. Depending on the purification strategy, the EV corona may be retained, altered, or stripped away entirely, yielding substantial variation in proteomic profiles and functional readouts.

In addition to compositional differences, EVs also carry active surface‐associated enzymes such as metalloproteinases that contribute to their functional identity [15]. Early evidence for enzymatic activity associated with EV membranes was provided by Fourcade et al. [16], who demonstrated that secretory phospholipase A2 (sPLA2) mediates lipid mediator generation on shed microvesicles. However, a systematic evaluation of metalloproteinases including ADAM10 activity normalized to the single EV level across isolation methods is lacking. Such enzymatic activities provide a unique, cell‐free readout of vesicle functionality, independent of uptake or cellular context. This raises a central question for translational EV research: are we analyzing vesicles as they exist in physiological contexts, or procedure‐shaped artefacts with altered biomedical utility?

In this study, we systematically compared four EV isolation strategies combining orthogonal characterization techniques, deep proteomic profiling, and functional assays targeting vesicle‐associated protease activity. By focusing on ADAM10 as a model enzyme, we demonstrate how enzymatic activity can serve as a direct tracer of EV functionality, complementing structural and compositional analyses. This integrated approach enables a comprehensive assessment of how isolation methods affect classical EV metrics, proteome composition, and enzymatic activity, providing a framework for evaluating workflows for analytical rigor in developing EV‐based diagnostics and therapeutic materials.

## 2 Materials and Methods

### 2.1 Cell Culture

ES‐2 cells were maintained at 37°C in 5% CO2 in Dulbecco's Modified Eagle Medium (Gibco, Thermo Fisher Scientific, Waltham, MA, USA) supplemented with 10% fetal bovine serum (Gibco, Thermo Fisher Scientific) with 1 % Pen/Strep. For EV production, 7 × 10^6^ cells were seeded per T175 flask (Greiner Bio‐One GmbH, Frickenhausen, Germany) and incubated at 37°C. After 24 h, the medium was replaced by CD293 medium (Gibco, Thermo Fisher Scientific) supplemented with 1% penicillin‐streptomycin and 1x GlutaMAX (Gibco, Thermo Fisher Scientific), followed by incubation for 48 h at 37°C under hypoxic conditions (5% CO2, 1% O2). CD293 medium is chemically defined and protein‐free, eliminating background proteins from the culture medium.

### 2.2 Ascites

Ascites fluid was collected from untreated high‐grade serous ovarian carcinoma patients undergoing first‐line surgery at the University Hospital Marburg (Marburg, Germany). The institutional ethics committee (reference number 205/10) approved the protocols by which informed consent was obtained from all patients. The cells were then subjected to a series of centrifugation steps. Initially, the cells were removed using centrifugation at 300 × g for 10 min at 4°C. This was followed by a second centrifugation step at 2500 × g for 10 min at the same temperature. Cell‐free ascites sera were cryopreserved at −80°C for subsequent use. For downstream analyses, pooled ascites samples were used.

### 2.3 Sample Preparation

Ascites fluid and ES‐2 cell culture supernatant were subjected to sequential centrifugation steps (500 × g, 5 min; 2000 × g, 10 min; 10 000 × g, 45 min; all at 4°C) to remove cells, debris, and large vesicles, respectively. Due to residual particulates in ascites, an additional 0.22 µm filtration step was performed. Cleared fluids were either processed immediately for EV isolation or stored at −80°C in 25 mL aliquots.

### 2.4 EV Isolation by UC‐UC

Cleared ES‐2 cell culture supernatants or ascites were transferred into 25 × 89 mm ultracentrifugation tubes (Beckman Coulter, Krefeld, Germany), tared with PBS, and centrifuged at 110 000 × g for 120 min at 4°C (SW32Ti rotor; Optima XPN‐80; Beckman Coulter). Supernatants were discarded, and pellets were resuspended in PBS by vortexing and incubated on ice for 5 min. For cell culture samples, pellets from six tubes (150 mL) were pooled to a final volume of 500 µL; for ascites, pellets from three tubes (75 mL) were combined similarly. To generate final UC‐UC samples, 500 µL of the concentrated material was diluted 1:20 in 1x PBS (total volume: 10 mL) and subjected to a second ultracentrifugation at 110 000 × g for 120 min at 4°C (14 × 98 mm tubes; SW41Ti rotor; Optima XPN‐80; Beckman Coulter). Pellets were resuspended in PBS following brief vortexing and 5 min incubation on ice. Final volumes were adjusted to 100 µL per sample. EV preparations were either stored at −80°C or used immediately for downstream analyses.

### 2.5 EV Isolation by UC‐DG

For iodixanol‐based density gradient ultracentrifugation, 0.5 mL of EV sample obtained from initial ultracentrifugation was carefully layered onto a discontinuous gradient composed of 5%, 10%, 20%, and 40% iodixanol in 14  mm × 98 mm ultracentrifugation tubes (Beckman Coulter). Gradients were centrifuged at 100 000 × g for 16–18 h at 4°C using an SW41Ti rotor (acceleration 5, deceleration off; Optima XPN‐80; Beckman Coulter). Twelve 1 mL fractions were collected from the top of each gradient and kept on ice. The density of each fraction was determined using a refractometer (Avantor, Radnor, PA, USA) and calculated based on a standard curve generated from iodixanol solutions. Fractions with densities between 1.071 and 1.123 g/mL were pooled and used for further processing, diluted 1:10 with 1x PBS, and centrifuged at 110 000 × g for 2 h at 4°C. Resulting EV pellets were resuspended in 1x PBS, incubated on ice for 5 min after brief vortexing, and pooled to a final volume of 100 µL. Samples were stored at −80°C or used immediately for downstream applications.

### 2.6 EV Isolation by TFF‐UF

Pre‐cleared ascites or ES‐2 supernatant was concentrated using a TFF‐Easy column (HansaBioMed Life Sciences Ltd., Tallinn, Estonia) operated with a peristaltic pump at 150 rpm according to the manufacturer's instructions (uniPeristalticpump3; LLG Labware, Meckenheim, Germany). In contrast to advanced TFF platforms, this setup did not provide active control of shear rates or real‐time monitoring of transmembrane pressure. The resulting 500 µL concentrates were transferred to Amicon Ultra centrifugal filters (100 kDa cutoff; Merck KGaA, Darmstadt, Germany) and centrifuged at 3000 × g and 4°C. Centrifugation times depended on sample viscosity (ES‐2: 4–8 min; ascites: 20–30 min). The retentate was recovered and transferred to 1.5 mL tubes. Final volumes were 100 µL (ES‐2) and 200 µL (ascites). Samples were stored at −80°C or used directly for downstream analyses.

### 2.7 EV Isolation by TFF‐SEC

TFF‐concentrated samples (500 µL) from ascites or ES‐2 supernatant were applied to self‐packed SEC columns (10 mL volume, Sepharose CL‐2B matrix, Cytiva, Marlborough, MA, USA) pre‐equilibrated with ≥20 mL 0.2 µm‐filtered 1xPBS. Fourteen 500 µL fractions were collected at room temperature and kept on ice. Particle‐rich fractions (typically 8–10, based on nanoparticle tracking analysis) were pooled, yielding 1.5 mL of eluate. To concentrate the EV‐containing eluate, pooled fractions were subjected to ultrafiltration using Amicon Ultra centrifugal filters (100 kDa cutoff; Merck) at 3000 × g and 4°C for 4–10 min, depending on sample viscosity. The final volume (100 µL) was recovered and stored at −80°C or processed immediately on ice.

### 2.8 Protein Concentration using BCA

Protein concentrations were measured using a Pierce BCA Protein Assay Kit (Thermo Scientific) according to the manufacturer's instructions.

### 2.9 Nanoparticle Tracking Analysis (NTA)

Particle concentration and size of each sample were determined using a ZetaViewer PMX110 (Particle Metrix, Inning am Ammersee, Germany). All samples were diluted in PBS to a final volume of 1 mL. Measurement concentrations were found by pre‐testing the ideal particle per frame value (100–300 particles/frame). For each measurement, three cycles were performed by scanning 11 cell positions each and capturing 30 frames per position with the following settings: Focus: autofocus; Camera sensitivity: 70; Shutter: 70; Scattering Intensity: detected automatically; Cell temperature: 25°C. After capture, the videos were analyzed by the in‐built ZetaView Software 8.05.05 SP2 with specific analysis parameters: Maximum area: 1000, Minimum area: 10, Minimum brightness: 25. Hardware: embedded laser: 40 mW at 488 nm; camera: CMOS.

### 2.10 Nano‐Flow Cytometry (nFC)

The particle concentration and size of each sample were determined via nFC with a NanoAnalyzer equipped with a 488 and 637 nm laser (NanoFCM Co., Ltd., Nottingham, UK). The cytometer was calibrated with 200 nm polystyrene beads (NanoFCM Co., Ltd.) at a concentration of 2.08 × 10^8^ particles/mL. Monodisperse silica beads of four different diameters (68, 91, 113, 155 nm) served as a size reference standard. The background signal was defined as the measurement of freshly filtered (0.1 µm) 1× TE buffer, which was subtracted from all subsequent measurements. For each measurement, data were collected for one minute with a sample pressure of 1.0 kPa. Each sample was diluted with 0.1 µm filtered 1× TE buffer to reach an optimal particle count range of 2500–12 000 events. The NF Profession V2.0 software was used to calculate the particle concentration and size distribution (NanoFCM Co., Ltd.). For immunofluorescence staining, the following antibodies were used (BioLegend, Koblenz, Germany): Alexa 488‐conjugated mouse anti‐human CD9 antibody (clone HI9a); APC‐conjugated mouse anti‐human CD63 antibody (clone H5C6); APC‐conjugated mouse anti‐human CD81 antibody (clone TAPA‐1); Alexa 488‐conjugated mouse anti‐human CD29 antibody (clone TS2/16). Isotype controls included FITC‐conjugated mouse IgG1, κ (clone MOCP‐21) at a concentration of 2 ng/µL in 100 µL 1× PBS. After centrifuging at 12 000× g for 10 min to remove antibody aggregates, the supernatant was added to 4 × 10^8^ purified EVs. The mixture was incubated overnight at 4°C with continuous shaking, followed by washing with 1 mL of filtered 1× PBS (0.22 µm) by ultracentrifugation at 110 000× g for 60 min at 4°C (Optima Max‐XP; TLA‐55 rotor; Beckman Coulter). The pellet was resuspended in 50 µL of 1× PBS for nFC analysis.

### 2.11 Transmission Electron Microscopy

Formvar‐coated copper grids (Science Services, München) were loaded with 5 µL of undiluted EV sample and incubated for 20 min at RT. Grids were blocked for 30 min using Aurion blocking solution (Aurion #905.001), then washed three times for 2 min in DPBS containing 0.1% BSA‐c (pH 7.4; Aurion, #900.099). Grids were fixed in 10% glutaraldehyde (Sigma, #G5882‐100ML) in DPBS for 5 min, followed by three washes in ddH_2_O for 2 min each. Negative staining was performed with 1.5% uranyl acetate (Agar Scientific, #R1260A) for 4 min before blotting. For each sample, an unstained control grid was prepared. Images were acquired using a Gatan OneView 4K camera on a JEM‐2100Plus (Jeol, Freising, Germany) at 200 kV and analyzed with ImageJ (National Institutes of Health—NIH).

### 2.12 Mass Spectrometry‐Based Proteomics

Extracellular vesicles (EVs) were isolated and collected in 50 µL of PBS buffer. To achieve complete lysis of the vesicles, 30 µL of 8% Sodium lauroyl sarcosinate (SLS, Sigma–Aldrich, Merck KGaA, Darmstadt, Germany) in a 50 mm Triethylammonium bicarbonate (TEAB, Sigma–Aldrich) buffer was introduced, resulting in a final SLS concentration of 3%. Samples were then subjected to incubation at 95°C for 10 min with shaking to facilitate lysis. Following lysis, protein reduction and alkylation were performed. First, dithiothreitol (DTT, Sigma–Aldrich) was added to a final concentration of 10 mm and incubated at 95°C for 10 min. This was followed by the addition of iodoacetamide (IAA, Sigma–Aldrich) to a final concentration of 13 mm, with incubation for 30 min in the dark at room temperature (RT).

For further sample processing, a modified SP3 method (Hughes et al., 2019) was employed using a custom‐made magnetic rack. Protein binding occurred in a 70% anhydrous acetonitrile (ACN, Chemsolute, Th. Geyer GmbH & Co. KG, Renningen, Germany) solution at a neutral pH, followed by washing with 70% ethanol (Roth GmbH, Karlsruhe, Germany) and then twice with 100% anhydrous ACN. After the ACN was removed, the beads were resuspended in 30 µL of 50 mm TEAB buffer, and 1 µg of trypsin (Promega, Madison, Wisconsin, USA) was added for protein digestion, which was carried out overnight at 37°C with shaking.

The sample volume was then reduced to approximately 5 µL using a SpeedVac concentrator (Eppendorf, Hamburg, Germany). Peptide binding to the beads was initiated by adding 100% ACN to achieve a final concentration greater than 95%. The beads were washed twice with the same solvent. Peptides were then eluted by adding 30 µL of 0.1% formic acid with 0.015% Dodecyl‐β‐D‐maltoside (DDM) and transferred into MS‐vials. The peptide concentration was estimated using the Pierce Quantitative Fluorometric Peptide Assay (Thermo Fisher Scientific), and sample volumes were adjusted to ensure equal concentrations.

The purified peptides were analyzed by liquid chromatography‐tandem mass spectrometry (LC‐MS) using a Bruker Daltonics timsTOF Ultra instrument coupled with a Bruker Daltonics nanoElute. Approximately 50 ng of peptides were loaded onto a C18 precolumn (Thermo Trap Cartridge 5 mm, µ‐Precolumn Cartridge / PepMap C18, Thermo Scientific). The peptides were then eluted in backflush mode through a reverse‐phase high‐performance liquid chromatography (HPLC) separation column (PepSep Ultra, C18, 1.5 µm, 75 µm × 25 cm, Bruker Daltonics) at a flow rate of 300 nL/min. The gradient elution program consisted of a gradual increase in Solvent B (99.85% acetonitrile and 0.15% formic acid), starting from 2% and rising to 17% over 36 min, then from 17% to 25% over 18 min, and finally from 25% to 35% over the last 6 min. The outlet of the analytical column was connected to the MS instrument via a CaptiveSpray 20 µm Emitter (Bruker Daltonics). Data acquisition utilized a data‐independent acquisition (DIA) method provided by Bruker. Precursor ion spectra were acquired with a fixed resolution of 45 000 and a mass range of 100–1700 m/z. Ion mobility ranged from a 1/k0 of 0.64 to 1.45 V s/cm^2^ with a 100 ms ramp time. This was followed by DIA scans with 24 fixed windows, each 25 m/z wide, covering a range from 400 to 1000 m/z.

Peptide spectrum matching and label‐free quantification were performed using DIA‐NN [17]. This software conducted a library‐free search against the Human Uniprot.org database (20429 reviewed Swiss‐Prot entries; January 2024), filtering the output to a 1% false discovery rate at the precursor level. An in silico spectral library was generated using deep learning to enable the library‐free search.

The search parameters included a fragment m/z range of 100–1700, and in silico peptide generation allowed for N‐terminal methionine excision and tryptic cleavage after K* or R*, with a maximum of one missed cleavage. Peptide lengths were restricted to a minimum of 7 and a maximum of 30 amino acids. Cysteine carbamidomethylation was set as a fixed modification, while methionine oxidation was considered a variable modification. Precursor masses from 100 to 1700 m/z and charge states one to four were evaluated, with a mass accuracy of 15 ppm for both MS1 and MS2 scans. The match‐between‐runs (MBR) algorithm was not employed.

Downstream data processing and statistical analysis were conducted using the in‐house Autonomics package (version 1.13.19, DOI:10.18129/B9.bioc.autonomics). Proteins with a q‐value of less than 0.01 were selected for further analysis. MaxLFQ values (Cox et al., 2014) were used for quantification, and missing values were imputed. The results were visualized using the R packages: ComplexUpset [18], UpSet [19], ggplot2 [20].

DIA‐NN initially identified 10 504 protein groups. All intensities and MaxLFQ values with only one precursor per sample were replaced with NA. After removing 2,833 protein groups without replication within a subgroup and filtering out 932 proteins identified with fewer than two peptides, 6,739 protein groups remained for analysis. The differential abundance of these protein groups was assessed by Autonomics using a Bayesian moderated t‐test, as implemented in the limma package (Ritchie et al., 2015). The full list of DIA‐NN settings can be found in the report.log.txt, and the full code for data processing and statistical analysis has been uploaded along with the mass spectrometric raw data to the ProteomeXchange Consortium with dataset identifier PXD069821 via the MassIVE partner repository (MassIVE‐ID: MSV000099567; DOI: 10.25345/C5PC2TP1N).

### 2.13 Western Blotting

Each EV sample group was normalized to 1 × 10^8^ particles, then added with 5x Laemmli buffer and 10x NuPAGE sample reducing reagent (Thermo Fisher Scientific). Blocking was performed with 5% milk powder in TBS‐T. Primary ADAM10 antibodies (1:2000, EPR5622, Abcam) and ADAM10 ectodomain primary antibody (1:1000, MAB1427, Biotechne), Flotillin (1:500, clone 18, BD Biosciences), ALIX (1:500, 1A12, Santa Cruz Biotechnology), and TSG101 (1:2000 AB1, Sigma–Aldrich) were diluted at different concentrations. After 1 h incubation with HRP‐coupled secondary antibodies and (1:10 000) 3x washing with TBS‐T, chemiluminescence was detected by adding WesternBright Sirius HRP substrate (K‐12043‐D20, Advansta, USA) and using the ChemiDoc MP Imaging System (Bio‐Rad Laboratories GmbH, USA).

### 2.14 FRET‐Based Activity Assays

To detect ADAM10 activity in EV preparations, a FRET‐based peptide based on PepDAB10 [21]; Dabcyl‐Ser‐Pro‐Leu‐Ala‐Gln‐Ala‐Val‐Arg‐Ser‐Ser‐Lys(5‐FAM)‐NH2; Biozyme Inc., Apex, NC; USA) was used. For time‐lapse fluorometry, a final substrate concentration of 10 µm (diluted from a 5  mm stock in DMSO) in 50 µl of activity buffer (1 µm ZnCl_2_, 20 mm Tris‐HCl pH 8.0, 10 mm CaCl_2_, 150 mm NaCl, 6 × 10%–4% Brij‐35) was incubated with 10^8^ EVs from different preparation methods in 96‐well microtiter white opaque plates (Sarstedt, Nümbrecht, Germany). All samples were run in technical triplicates. Assays were performed in a multi‐well plate reader (Fluostar Optima, BMG Labtech, Offenburg, Germany) with excitation wavelength of 485 nm and an emission of 530 nm. Activities were monitored as fluorescence units every two minutes. For inhibition assays, a specific ADAM10 Inhibitor (GI254023X, Sigma–Aldrich, Dreieich, Germany) and a broad‐range MMP/ADAM Inhibitor Batimastat (BB‐94, Sigma–Aldrich, Dreieich, Germany) were used, respectively.

### 2.15 Trans‐Shedding of CD23 by ADAM10 in EVs

HEK293 T cells stably expressing CD23 were generated as described earlier (Schlomann et al., 2019 https://doi.org/10.1515/hsz‐2018‐0396). To test EV preparations from ES‐2 cells for their ADAM10 activity to release soluble CD23 (sCD23), 100.000 cells were seeded in each well of a 24 well plate. After overnight incubation, medium was changed to 500 microlitres of medium containing 1.3 × 10^9 EV from different preparations or control medium. After 24 h of incubation with EVs, supernatants were collected, centrifuged at 13.000 x g for 10 min and subjected to a CD23 ELISA (R&D Systems, Biotechne (DY123) using a 1:100 dilution. For inhibitor controls, 150 microM of GI254023X and 50 microM of Batimastat (BB‐94) were used, respectively.

### 2.16 Statistics

Statistical analyses were performed using GraphPad Prism 10 and RStudio (version 2024.12.1). One‐way ANOVA with Tukey's post hoc test was used for normally distributed data; non‐parametric data were analyzed using the Kruskal‐Wallis test followed by Dunn's test with Bonferroni correction. Data are shown as mean ± SD or as box‐and‐whisker plots (min‐max) with individual replicates.

## 3 Results

### 3.1 Isolation Workflows Impose Distinct Proteomic Signatures Beyond a Shared EV Core

**FIGURE 1:**
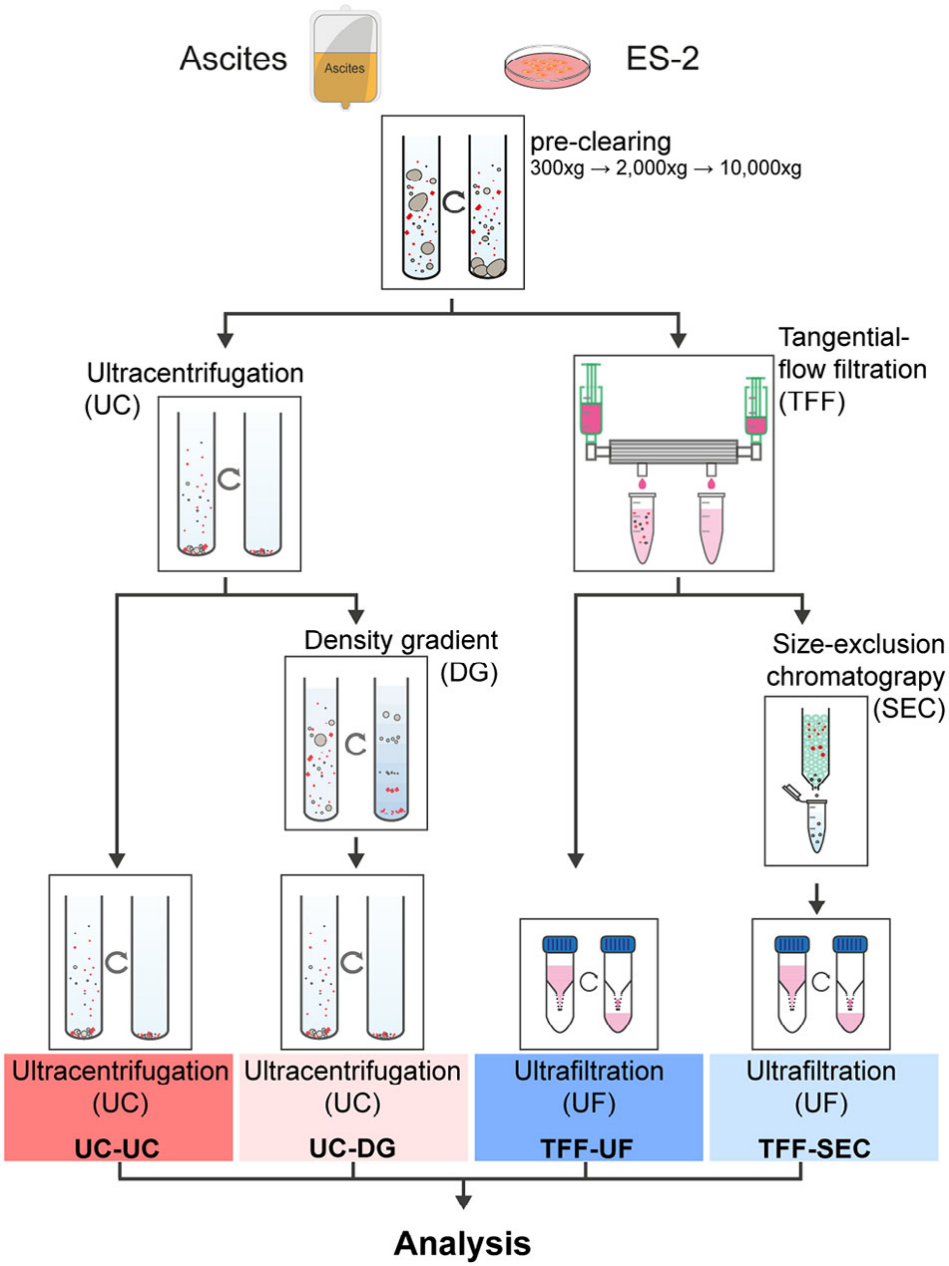
Workflow for EV isolation from ascites and ES‐2 cell culture supernatant. Cell‐free ascites and conditioned medium from ES‐2 ovarian cancer cells were subjected to sequential centrifugation (300 × g, 2000 × g, 10 000 × g) to remove cells, debris, and large vesicles. Pre‐cleared fluids were processed by one of four EV isolation strategies: ultracentrifugation (UC‐UC), ultracentrifugation followed by density gradient (UC‐DG), tangential flow filtration followed by ultrafiltration (TFF‐UF), or TFF followed by size‐exclusion chromatography (TFF‐SEC). Final EV preparations were concentrated to ∼100 µL and used for multi‐modal characterization, including mass‐spectrometry‐based proteomics.

**FIGURE 2:**
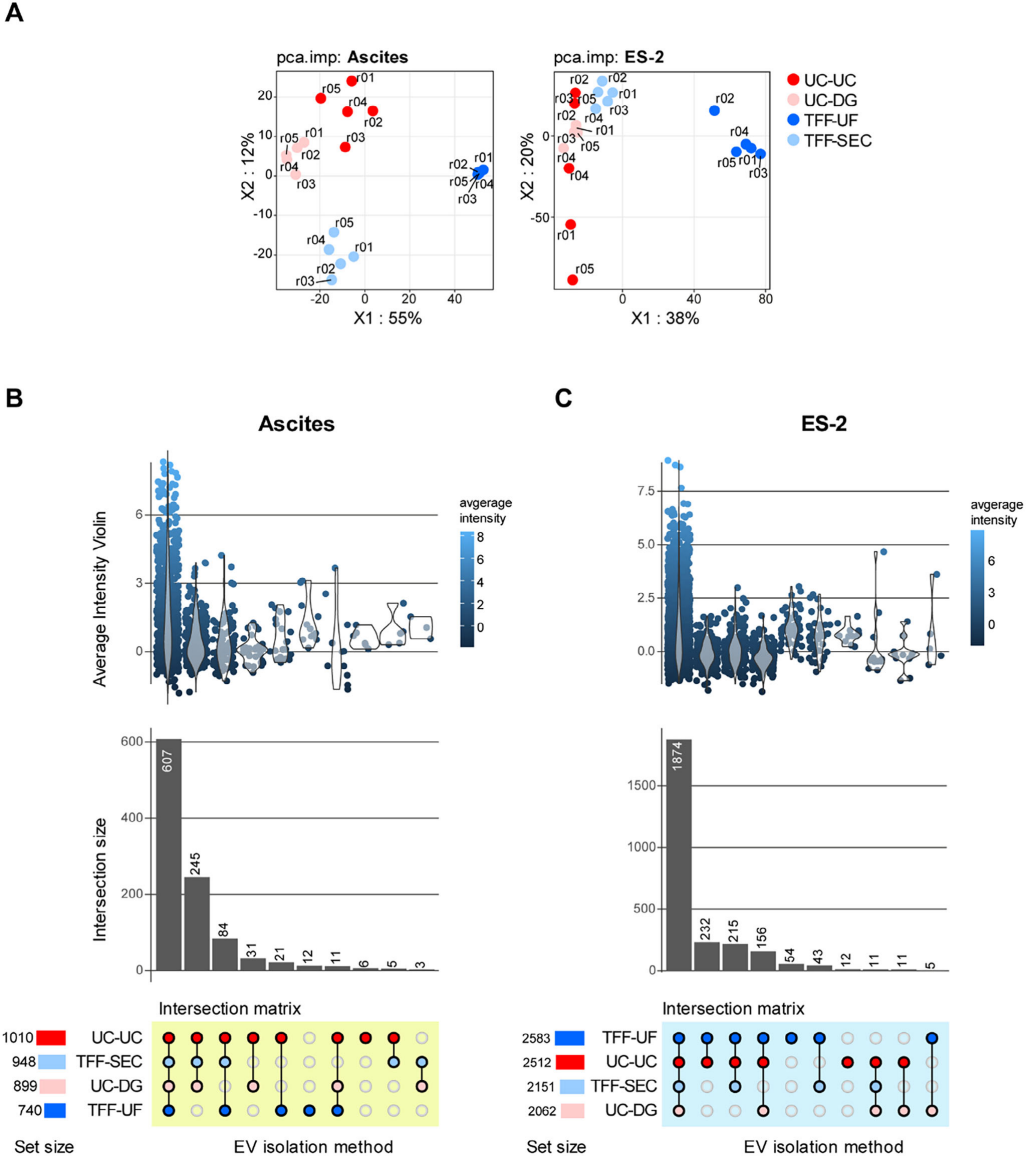
Proteomic separation of EVs by isolation method. (A) PCA biplot of log2‐transformed intensities. Axis labels indicate the percentage of variance explained by PC1 and PC2. Proteins driving separation along each component are highlighted in the bottom panels. (B) ES‐2‐derived EVs: UpSet plot showing protein group intersections across workflows (restricted to proteins detected in ≥3/5 replicates per condition). Distribution plots compare average intensities of shared versus method‐specific subsets. (C) Ascites‐derived EVs: same analysis as in (B).

EVs were isolated using four approaches: UC‐UC, UC‐DG, TFF‐UF, and TFF‐SEC (Figure 1) from ascites fluid (n = 6 donors) and serum‐free, chemically defined ES‐2 cell culture supernatants (8 biological replicates). To assess how isolation workflows shape EV identity, we examined the global proteomic profiles of these preparations by label‐free quantitative mass spectrometry with protein input digestion normalized to 2 × 10^8^ particles per sample (measured by nFC). Unsupervised principal component analysis (PCA) of normalized log_2_‐transformed intensities revealed the dominant source of variance in both datasets. In ascites EVs, PC1 (49% total variance explained) clearly separated UC‐UC from the other methods, while PC2 (16%) reflected variability among TFF‐ and DG‐based preparations (Figure 2A). A comparable pattern was observed in ES‐2 EVs, where PC1 (55%) again distinguished UC‐UC, and PC2 (12%) captured additional variation. Distinct protein subsets contributed to the separation along both components, including markers linked to vesicle biogenesis, membrane trafficking, or non‐vesicular contaminants, underlining that isolation‐dependent signatures were not solely driven by technical noise.

Intersection analysis quantifying the overlap and interconnectedness of protein detection across methods revealed that 1,874 proteins in ascites and 607 proteins in ES‐2‐derived EVs were consistently detected in all samples (Figure 2B,C). This finding reflects the broader proteomic coverage achievable in cell line‐derived preparations compared to the inherently more complex ascites samples. The relative abundance of overlapping proteins varied across purification methods, whereas qualitative differences ‐namely method‐specific proteins‐ were comparatively low. This effect was particularly evident in ascites‐derived proteomes, likely reflecting their broader dynamic range. Collectively, these findings define a core protein signature in ES‐2 and ascites EVs consistently captured across all methods, while the isolation strategy primarily influenced detection levels with only moderate qualitative differences.

### 3.2 MISEV Categories Expose Selective Enrichment Across Workflows

To systematically compare the EV protein profiles across isolation methods, all identified proteins were annotated according to a reference list based on the MISEV2023 guidelines [22] supplemented with EV‐associated proteins recently defined [23] and lipoprotein‐associated proteins, exomers, and mitochondrial components (Table S1). Each comprised protein was assigned one of five categories: 1) transmembrane or GPI‐anchored proteins of plasma membrane/endosomes; 2) cytosolic proteins recovered in EVs; 3) major components of non‐EV co‐isolated structures (lipoproteins, exomers, supermers); 4) lipid‐bound, and soluble proteins associated with intracellular compartments other than plasma membrane/endosomes; 5) secreted and corona proteins.

**FIGURE 3:**
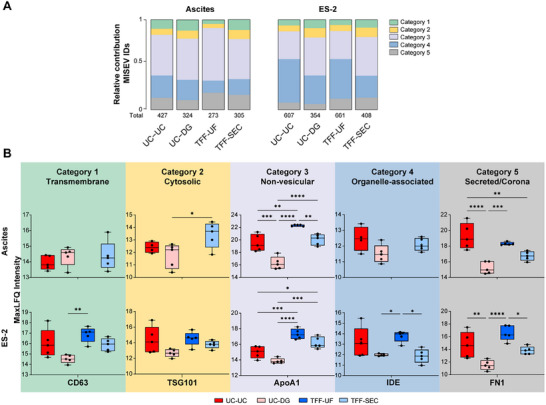
Differential protein category composition and marker detection in EV preparations obtained by four isolation methods. (A) Relative peptide abundance (%Np) of EV proteins categorized according to MISEV2023 criteria. Proteins were grouped into five categories: **Category 1** (green): transmembrane; **Category 2** (yellow): cytosolic; **Category 3** (gray): non‐vesicular; **Category 4** (blue): organelle‐associated; **Category 5** (red): secreted/corona‐associated proteins. Values are shown for each isolation method—UC‐UC, UC‐DG, TFF‐UF, and TFF‐SEC—applied to EVs from either ascites or ES‐2 cell culture supernatants (n = 5 per condition). (B) Detection of representative proteins from each category across the four isolation methods based on MaxLFQ intensities since the global signal delivered did not strongly differ among methods. Abundances are visualized using boxplots (mean ± SEM) for each method: UC‐UC (dark red), UC‐DG (light red), TFF‐UF (dark blue), and TFF‐SEC (light blue). Asterisks indicate significant differences between methods (* < 0.05, ** < 0.01, *** < 0.001, **** < 0.0001; two‐way ANOVA with Tukey's multiple comparisons test).

The distribution of these categories varied according to isolation method (UC‐UC, UC‐DG, TFF‐UF, TFF‐SEC) and sample source (ascites, ES‐2) (Figure 3A). UC‐DG and TFF‐SEC consistently showed the highest relative enrichment of canonical EV proteins (categories 1 and 2). In contrast, TFF‐UF displayed higher proportions of co‐isolates and secreted proteins (categories 3 and 5), while UC‐UC yielded an intermediate profile with increased intracellular proteins (category 4) in ascites‐derived samples. ES‐2‐derived EVs generally contained fewer non‐EV proteins than ascites preparations, reflecting matrix‐specific background differences.

Targeted evaluation of representative markers supported these trends (Figure 3B; Figure S1). Canonical EV markers (CD63, CD81, ALIX, TSG101; categories 1–2) were enriched in UC‐DG and TFF‐SEC, whereas non‐vesicular proteins such as ApoA1 were predominantly detected in TFF‐UF. UC‐UC contained medium to high levels of EV markers. The limited EV‐marker detection observed in TFF‐UF might be due to highly abundant serum proteins (e.g., albumin, ApoA1), which limit the dynamic detection range. Method‐specific differences were also evident for category 2b proteins (HSP70, tubulin): both were detected in UC‐UC and TFF‐UF, but strongly reduced in UC‐DG and TFF‐SEC, particularly in ascites‐derived EVs. TFF‐UF was dominated by category 3 proteins, while UC‐UC frequently contained category 4 markers, including mitochondrial and secretory proteins. By contrast, TFF‐SEC showed a balanced profile with strong EV marker representation and minimal contamination.

Overall, MISEV‐based annotation considering categorical distribution demonstrated that UC‐DG and TFF‐SEC yielded the most marker‐enriched and least contaminated EV proteomes, underscoring their suitability as preferred strategies for generating biochemically robust EV datasets.

### 3.3 EV‐Defining Proteins Highlight Method Performance

**FIGURE 4:**
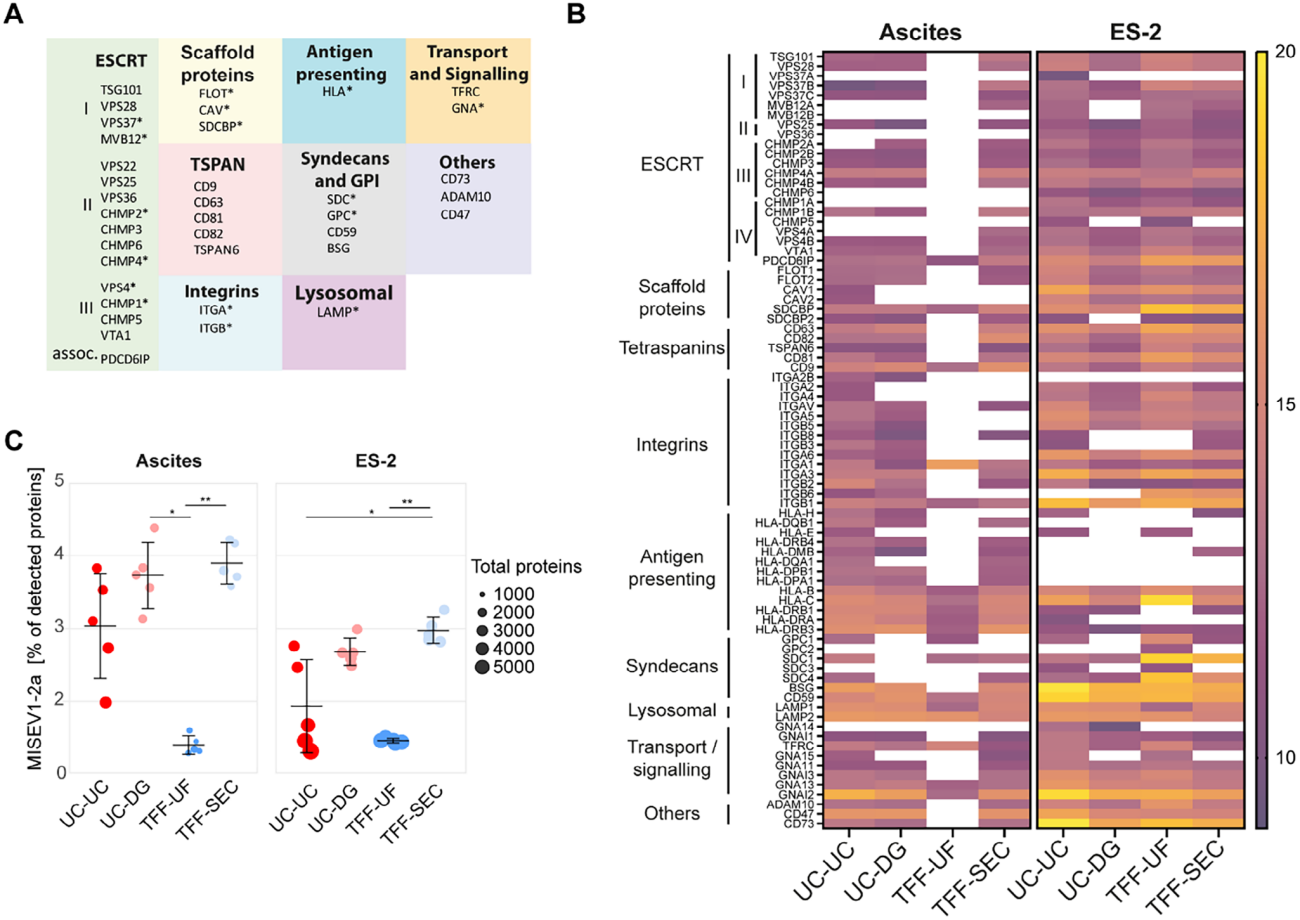
Profiling of canonical EV proteins (MISEV2023 categories 1 and 2a) across different isolation strategies. (A) Functional grouping of 118 proteins assigned to MISEV2023 categories 1 (transmembrane and lipid‐anchored proteins) and 2a (cytosolic proteins with membrane association), based on UniProt annotations. Proteins were categorized by biological function, including ESCRT components, membrane scaffolding proteins, tetraspanins, integrins, antigen‐presenting molecules, lysosomal markers, and transport/signaling proteins. Protein families are denoted by asterisks (e.g., ITGA* for integrin alpha chains). (B) Heatmap showing detection of 94 out of the 118 assigned proteins across EVs isolated from ascites and ES‐2 conditioned medium using four methods: UC‐UC, UC‐DG, TFF‐UF, and TFF‐SEC. Color intensity reflects relative abundance; absence of color indicates non‐detection. (C) Dot plots show the percentage of MISEV2023 category 1/2a proteins per replicate for each EV isolation method. Ascites‐ and supernatant‐derived samples are shown separately. Black bars indicate mean ± SD; point size reflects total protein count. Statistical differences between methods were assessed using Dunn's test with Bonferroni correction (* < 0.05; ** < 0.01).

To assess the quality and specificity of isolated EV fractions in greater detail, we compiled proteins from categories 1 and 2a of the MISEV2023 guidelines, representing structurally embedded membrane proteins and factors involved in vesicle biogenesis. This list comprises 118 EV markers, further grouped into nine functional categories: ESCRT, membrane scaffolding, tetraspanins, integrins, antigen presentation, syndecans, lysosomal proteins, transport/signaling, and others (Figure 4A; Table S1). Overall, 68 markers were detected across ascites‐ and ES‐2‐derived preparations. UC‐DG and TFF‐SEC consistently recovered the largest number of markers in both sources, whereas UC‐UC performed less efficiently (Figure 4B). Marker abundance was generally higher in EVs derived from cell culture media compared to ascites (Figure 4C). TFF‐UF showed the lowest detection rate in ascites, with 50 of the 68 markers not detected. Quantitative comparison of category 1 and 2a proteins confirmed that UC‐DG and TFF‐SEC achieved the highest marker detection rates, whereas TFF‐UF showed reduced recovery, particularly in ascites (Figure 4C). In addition, we evaluated known and putative ovarian cancer biomarkers in EV preparations, which also displayed method‐dependent recovery patterns (Figure S5).

Analysis of EV‐specific category 1 and 2a proteins demonstrated superior marker recovery with UC‐DG and TFF‐SEC, while TFF‐UF yielded the poorest performance, especially for ascites‐derived EVs.

### 3.4 Particle Yield and Size Distributions Vary by Workflow

**FIGURE 5:**
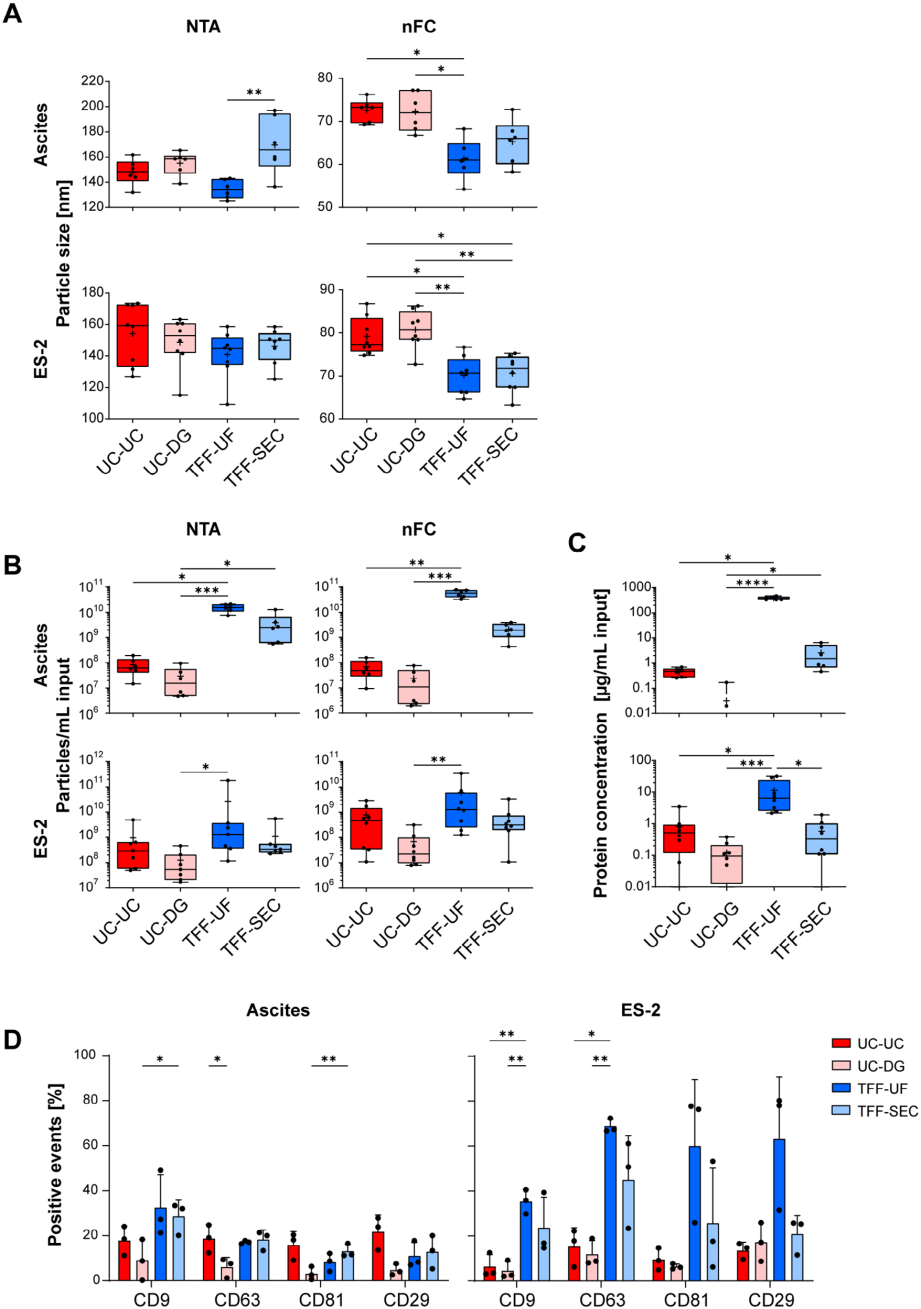
Quantitative and biophysical characterization of EVs isolated by four methods. (A) Particle concentrations from ascites (n = 6) and ES‐2 supernatant (n =  8) as determined by NTA and nFC. (B) Median particle diameters measured by NTA and nFC for the same samples and isolation strategies (UC‐UC, UC‐DG, TFF‐UF, TFF‐SEC). Y‐axis is presented on a log_10_ scale (C) Protein concentration assessed by BCA assay, shown as absolute values (ng/µL) and normalized per mL input. The y‐axis is presented on a log_10_ scale. Data are shown as box‐and‐whisker plots with whiskers extending to the minimum and maximum values, and all individual data points are plotted; center line indicates the median; boxes represent the interquartile range, whiskers denote minimum and maximum values, and “+” marks the mean. (**D)** EV marker analysis (CD9, CD63, CD81, CD29) in ascites‐ and ES‐2‐derived EVs across isolation methods. Statistical differences were assessed by one‐way ANOVA (*p < 0.05; **p < 0.01; ***p < 0.0005; ****p < 0.0001).

To complement these molecular benchmarks with physical characterization, we next compared particle size distributions and yields using NTA and nFC. For ascites‐derived EVs, NTA revealed the largest particles and highest variability with TFF‐SEC, whereas TFF‐UF yielded the smallest vesicles (Figure 5A; Figure S2). UC‐based methods showed lower variability, with UC‐DG yielding larger particles than UC‐UC. NTA reported particle sizes of 120–200 nm, while nFC consistently detected smaller particles (50‐80 nm). Across methods, UC produced larger vesicles than TFF, with minimal differences between TFF‐UF and TFF‐SEC.

For ES‐2‐derived EVs, NTA indicated particle sizes of 140–160 nm across all methods. Despite variability, mean sizes (60–85 nm) did not differ significantly. UC‐UC and UC‐DG produced larger vesicles than TFF‐based methods, while TFF‐UF and TFF‐SEC yielded comparably smaller particles.

The particle concentration was markedly higher (1‐2 orders of magnitude) in TFF‐based isolations compared to UC for ascites, and these methods also showed the highest yield in ES‐2‐derived EVs (Figure 5B). UC‐DG yielded the lowest recovery, and UC‐UC and TFF‐SEC produced intermediate yields. Both NTA and nFC showed comparable relative results, with nFC measurements consistently producing slightly higher values.

The protein concentration was determined by BCA assay and normalized to 1 mL of starting material to account for differences in input volume and concentration steps (Figure 5C). Overall, there was a strong correlation between particle concentration and protein content. In ascites‐derived samples, TFF‐based methods outperformed UC‐based protocols. Among these, TFF‐UF achieved the highest protein recovery, followed by TFF‐SEC, whereas UC‐UC and UC‐DG preparations generally remained below the detection limit. In ES‐2 supernatants, only TFF‐UF consistently yielded measurable protein above the detection threshold. Thus, in both sample types, TFF‐UF significantly outperformed all other methods, particularly UC‐DG, in terms of protein recovery.

Since protein content is frequently used to normalize EV input for downstream applications such as functional assays or omics analyses, these differences in protein yield across isolation strategies are highly relevant, emphasizing the need for accurate quantification and compatible assay sensitivity when comparing isolation efficiencies.

Next, the molecular phenotypic profile of EVs was assessed across isolation methods. EV preparations were stained for the canonical EV markers CD9, CD63, CD81, and CD29, and analyzed by nFC using 2 × 10^8^ particles per marker (Figure 5D; Figure S3). In ascites‐derived samples, UC‐DG consistently yielded the lowest proportion of marker‐positive events (<10%). All other purification strategies showed higher detection rates, with CD9 reaching up to 30% in TFF‐based methods. Significant differences between methods were detected for CD9, CD63, and CD81, particularly when comparing UC‐DG to TFF‐based protocols. In ES‐2‐derived EVs, TFF‐based methods outperformed UC‐based protocols across all markers, with TFF‐UF yielding the highest marker positivity.

Taken together, isolation methods differentially affected EV yield, size, protein co‐isolation, and marker detection. For example, TFF‐UF maximizes particle recovery and marker detection in ES‐2 supernatants, while the high protein concentration suggests that non‐EV proteins may also be co‐enriched. Similarly, for ascites‐derived samples, the benefits of each method were dependent on the specific metric used rather than consistent across all parameters.

### 3.5 Morphology Confirms EVs, While Background Patterns Diverge

**FIGURE 6:**
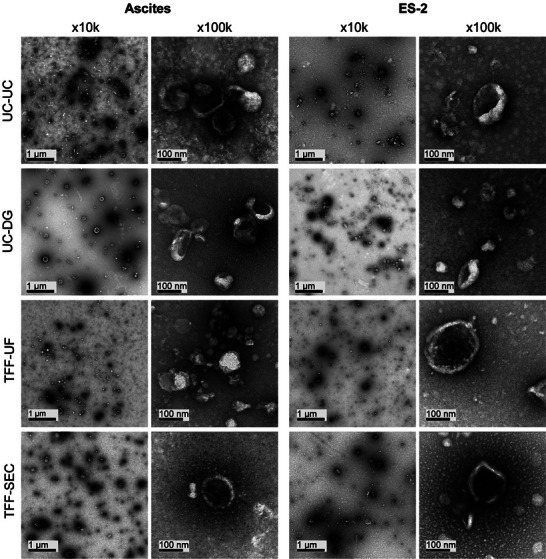
Transmission electron microscopy of EVs isolated by four different methods. Representative TEM images of EVs isolated from ascites and ES‐2 supernatant using UC‐UC, UC‐DG, TFF‐UF, and TFF‐SEC. For each condition, two magnifications are shown: ×10 000 (scale bar = 1 µm) and ×100 000 (scale bar = 100 nm). Images illustrate particle morphology and purity across isolation strategies and sources.

To further assess the integrity and morphology of EVs obtained from different isolation methods, representative preparations from ascites and ES‐2 supernatants were analyzed by transmission electron microscopy (TEM) (Figure 6). Across all methods, membrane‐bound, cup‐shaped particles consistent with EV morphology were observed, as expected from dehydration and negative staining procedures.

In ascites‐derived samples, characteristic EV‐like structures were readily identifiable at 10k magnification in UC‐DG and TFF‐SEC preparations. In contrast, TFF‐UF samples contained few vesicular structures, even at higher magnification (100k), and were dominated by heterogeneous, electron‐dense background material and non‐vesicular features. UC‐UC preparations contained EV‐like particles together with a considerable proportion of irregular, dense, or amorphous structures not consistent with classical vesicular morphology.

In ES‐2‐derived samples, vesicle‐like particles were detectable across all isolation methods, though small amounts of background material or non‐vesicular structures were present, particularly in UC‐UC preparations. TFF‐SEC and UC‐DG samples showed the most typical vesicular structures with low levels of potential contaminating features.

TEM is a qualitative method, with particle appearance influenced by sample preparation and staining procedures, the data thus do not allow any quantification, and representative examples are shown. Nevertheless, our observations indicate method‐dependent differences in sample purity and vesicle preservation. This further supports the notion that the isolation strategy profoundly affects both the quantitative and qualitative characteristics of EV preparations.

### 3.6 ADAM10 Activity Provides a Particle‐Normalized Functional Readout of EVs

**FIGURE 7:**
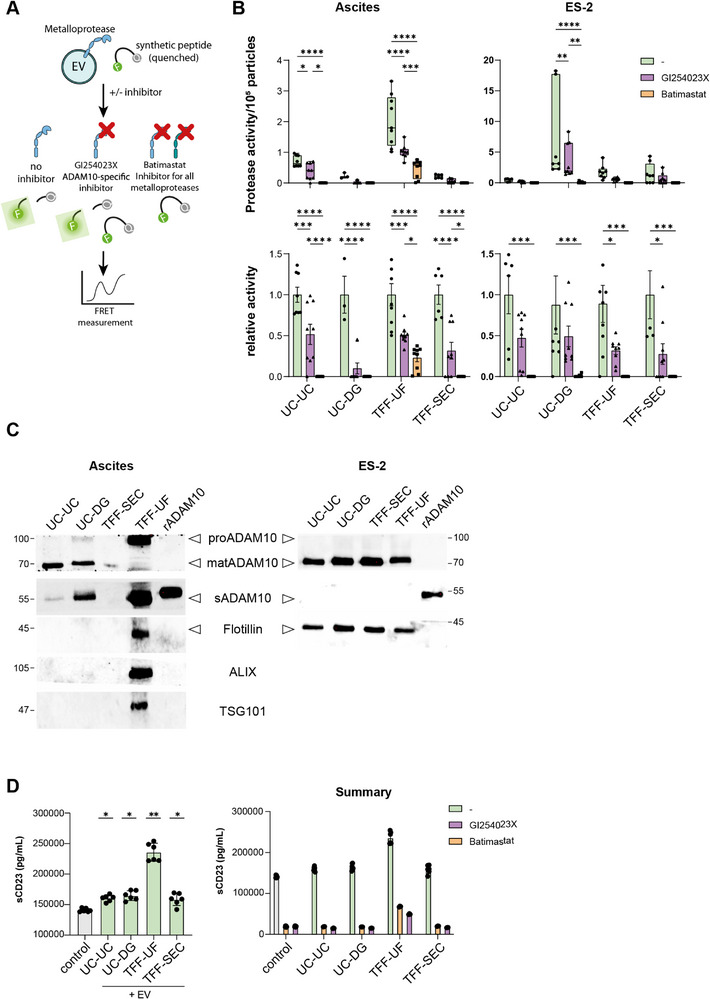
EV‐associated protease activity and inhibitor response across four isolation methods. (A) EV‐associated metalloprotease activities were assessed by cleavage of a FRET‐based fluorogenic peptide substrate. Proteolytic cleavage releases fluorescence from quenching. Samples were measured in real‐time (every 2 min) without inhibitor or after pre‐incubation with GI254023X (ADAM10‐specific) or Batimastat (pan‐metalloprotease inhibitor). (B) *Upper panel*: Quantification of absolute protease activity in EV preparations, normalized to 1 × 10^5^ particles to account for input differences. ***Lower panel***: Relative activity normalized to the no‐inhibitor control to assess inhibitor sensitivity. Data are shown as box‐and‐whisker plots (min to max) with all individual data points (n = 9). (C) Different forms of ADAM10 were detected in EVs isolated from either ES‐2 cell culture supernatant (n = 3) or ascites of ovarian cancer patients (n = 3) by four different methods and ADAM10 was detected either with antibody EPR5622 with an epitope located in the cytoplasm (pro and matADAM10), or with antibody MAB1427 with an epitope in the extracellular portion of ADAM10, thereby detecting the soluble form of ADAM10 (sADAM10). For all samples, 1 × 10^8^ EVs were loaded, except for Ascites‐TFF‐UF (5 × 10^7^ vesicles). 60 ng of rhADAM10 was used as a positive control for soluble ADAM10. EV markers (Flotillin, ALIX, TSG101) were below the detection level under these conditions, except for TFF‐UF ascites preparations, consistent with their higher protein yield. Molecular weight markers are indicated in kDa. D) ELISA assay to measure cleaved (soluble) CD23 protein released from CD23 expressing HEK293 cells without (left panel) and in the presence of inhibitor controls, 150 microM of GI254023X and 50 microM of Batimastat (BB‐94) (right panel). Statistical analysis: two‐way ANOVA with Tukey's post hoc test. * < 0.05; ** < 0.01; *** < 0.001; ****< 0.0001.

Given that ADAM10 is variably detected across workflows, we next asked whether vesicle‐associated functional activity scales with proteomic recovery, we established a vesicle‐based protease assay. Protease activities were measured using a cell‐free, enzyme‐based FRET assay with a quenched synthetic peptide substrate pepDAB10 (Figure 7A; Figure S4). This approach avoids common limitations of cell‐based assays, such as variability in uptake, receptor expression, or EV‐to‐cell ratios, and enables a direct, particle‐normalized readout of vesicle‐associated proteolytic activity.

In ascites‐derived EVs, TFF‐UF exhibited the highest activity, followed by UC‐UC, while UC‐DG and TFF‐SEC preparations yielded markedly lower levels (Figure 7B, **upper panel**). In contrast, for ES‐2‐derived EVs, UC‐DG displayed the strongest activity, with TFF‐UF and TFF‐SEC yielding intermediate levels, and UC‐UC the lowest (Figure 7B, **lower panel**). Broad‐range inhibition of metalloprotease activities using BB‐94 (Batimastat) abolished activity in all samples except for ascites EVs from TFF‐UF, suggesting the presence of additional proteases in this fraction.

To assess specifically the extent of ADAM10 activity, samples were treated with the specific inhibitor GI254023X [24]. In ascites EVs, inhibition reduced activity by ∼80% in UC‐DG, ∼70% in TFF‐SEC, and ∼50% in UC‐UC, while TFF‐UF showed only partial inhibition. In ES‐2 EVs, the strongest inhibition was observed in TFF‐SEC and UC‐DG, with UC‐UC showing little effect, consistent with greater protease heterogeneity.

Western blotting confirmed the presence of ADAM10 in all ES‐2 preparations, primarily as the active membrane‐bound form (Figure 7C). In ascites EVs, ADAM10 was detected in all methods, with TFF‐UF showing the highest abundance, in line with activity measurements. Soluble ADAM10 was present in all preparations except TFF‐SEC. In order to confirm that EV‐associated ADAM10 is enzymatically active in vitro and can recognize and shed the selected target CD23 on recipient cells, HEK293 target cells expressing membrane‐bound CD23 were incubated with EV preparations derived from ES‐2 cells. Released (soluble) CD23 in the supernatant was quantified by ELISA. All EV preparations from ES‐2 cells induced cleavage of membrane‐bound CD23 from HEK293 cells, demonstrating functional CD23 shedding activity of EV‐associated ADAM10 from cells. Notably, the highest cleavage rate was observed for EVs isolated by TFF‐UF (Figure 7D, left panel). CD23 release was strongly reduced upon treatment with the ADAM10‐selective inhibitor GI254023X (150 µ) and with the broad‐spectrum metalloprotease inhibitor Batimastat (BB‐94; 50 µm), confirming that CD23 cleavage was mediated by ADAM10/MMP activity associated with the EV preparations (Figure 7D, right panel).

### 3.7 Integrated Comparison of EV Isolation Strategies

**FIGURE 8:**
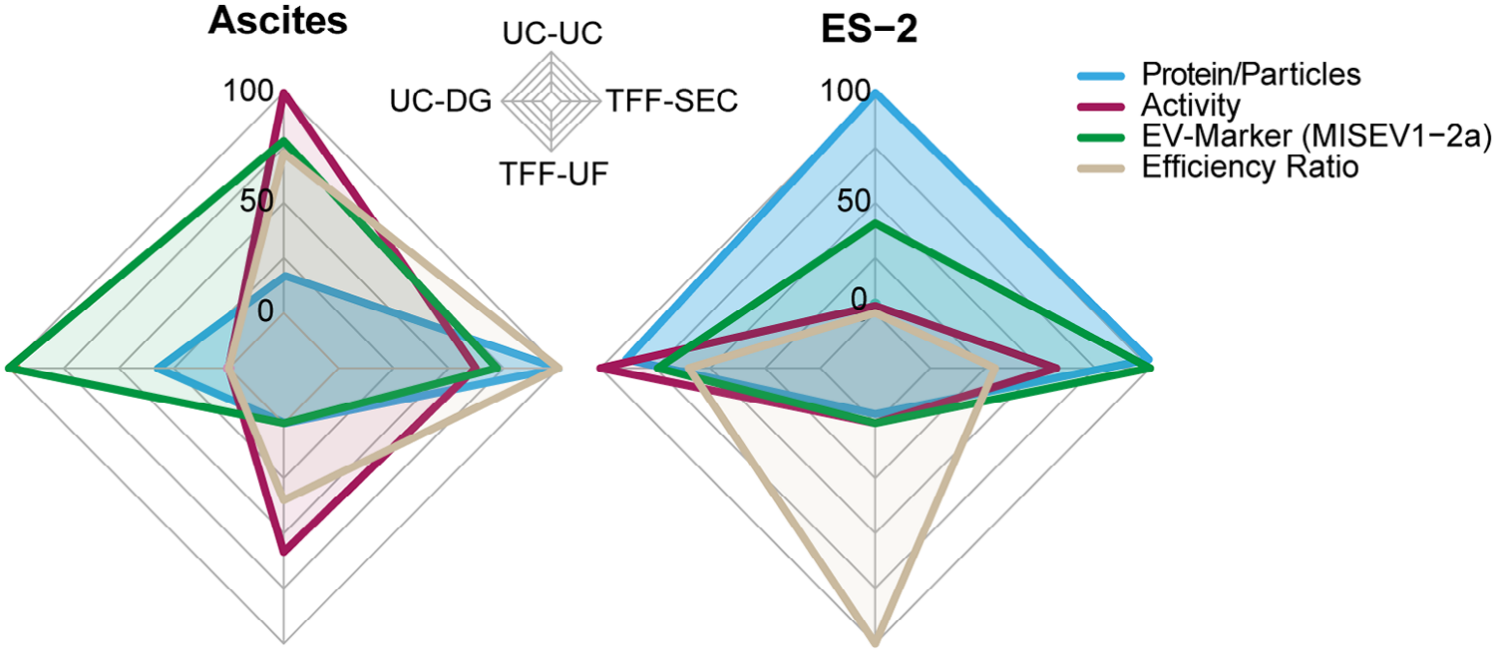
Integrated comparison of EV isolation strategies. Radar plots summarizing protein‐to‐particle ratios, protease activity, EV marker detection (MISEV1‐2a), and an efficiency index (particle recovery relative to canonical EV marker positivity by nFCM) for EVs isolated from ascites (left) and ES‐2 supernatants (right).

To integrate the multi‐dimensional outcomes of our comparative analysis, we summarized all key readouts in a radar plot (Figure 8). This visualization condenses protein‐to‐particle ratios, vesicle‐associated protease activity, canonical EV marker detection (MISEV1‐2a), and an efficiency index (relating particle recovery to the proportion of canonical EV marker‐positive events for CD9, CD63, CD81, and CD29 detected by nFCM) into a single comparative framework. By juxtaposing these parameters, the plot highlights method‐ and source‐specific strengths and weaknesses in an intuitive manner. UC‐DG purification from ascites provides very good recovery of MISEV proteins, indicating low contamination with non‐EV proteins. However, the yield and specific activity of the purified EVs are so poor that the method is not generally applicable. The situation differs for cell culture supernatant, where UC‐DG achieves high scores in all categories, including yield, activity, and purity. Overall, the high protein‐to‐particle recovery and activity observed with TFF‐UF are contrasted by reduced marker enrichment for both sources. Importantly, the integrated analysis underscores that no single strategy uniformly outperforms across all criteria, and that method‐aware interpretation remains essential for drawing biologically meaningful conclusions from EV datasets.

## 4 Discussion

Our comparative analysis demonstrates that isolation workflows do not merely differ in technical efficiency but actively shape the apparent EV proteome and, critically, the functional activities detected. This finding highlights that methodological choices significantly impact biological interpretation. While the MISEV (Minimal Information for Studies of Extracellular Vesicles) guidelines, established by the International Society for Extracellular Vesicles (ISEV), provide an essential framework for standardizing EV research and transparent reporting [22], they primarily focus on recovery of markers and the co‐isolation of contaminants. Less attention has been given to how isolation strategies redefine vesicle composition and, consequently, affect functional output.

Our data demonstrate that this impact is substantial: workflow choice not only dictates which proteins are detected but also whether critical enzymatic activities, such as ADAM10, are preserved or lost—thereby directly influencing the translational potential of EV preparations for biomarker discovery and therapeutic development.

In this study, we systematically compared four EV isolation workflows: differential UC (UC‐UC), density gradient UC (UC‐DG), TFF followed by ultrafiltration (TFF‐UF), and TFF followed by SEC (TFF‐SEC) (Figure 1) using comprehensive downstream analysis addressing differences in yield, EV size, the protein cargo, and vesicle‐specific protease activity.

Isolation by TFF‐SEC and TFF‐UF generally resulted in higher particle yields, particularly in ascites‐derived EVs, where particle numbers exceeded those obtained by ultracentrifugation by two orders of magnitude. This observation may reflect higher recovery efficiencies of TFF‐based methods but could also indicate co‐isolation of non‐vesicular particles. In line with this, protein‐to‐particle ratios are frequently used as a proxy for EV purity. However, high ratios may primarily reflect co‐isolation of soluble or lipoparticle‐associated proteins rather than increased EV content, particularly in protein‐rich matrices such as ascites, whereas low ratios obtained by UC‐DG reflect higher stringency at the expense of yield. Accordingly, UC‐DG produced the lowest particle counts yet yielded comparatively larger EV populations, supporting its established role as a stringent purification method. Interestingly, UC‐UC and TFF‐SEC produced intermediate yields, suggesting that these approaches may offer a balance between recovery and purity depending on the downstream application.

Such trade‐offs are not only technical but also application‐dependent, as recovery versus purity differentially impacts the suitability of EVs for functional assays, diagnostic pipelines, or therapeutic use.

A key novel aspect of our study is the functional comparison of vesicle‐associated protease activities after density‐ and size‐based purification. To the best of our knowledge, this represents the first systematic comparison of cell‐free, functional enzymatic readouts across isolation workflows under particle‐normalized conditions. Importantly, the workflow‐dependent differences observed in the particle‐normalized, cell‐free protease assay were also reflected at the level of a biologically relevant, cell‐surface substrate. EV‐mediated cleavage of the physiological ADAM10 target CD23 therefore provides a functional link between isolation‐dependent biochemical activity and downstream cellular effects. While yield and purity have been extensively evaluated, functional assays following gradient‐ or SEC‐based purification remain largely unexplored [4, 25].

Functionally, both the EV source and the isolation method influenced protease profiles. In ascites‐derived EVs, TFF‐UF exhibited the strongest activity, whereas in ES‐2 EVs, UC‐DG was most active, when measuring cleavage of synthetic peptides. Interestingly, in a cell‐based trans‐shedding assay measuring protease‐mediated cleavage of the surface receptor CD23, ES‐2 EVs isolated by TFF‐UF induced higher cleavage activity than UC‐DG. This indicates that functional readouts can diverge from marker‐based purity, underscoring a direct link between isolation workflow and EV‐mediated proteolysis. The stringent purification does not abolish vesicle functionality but can preserve biologically relevant enzymatic activities, potentially influenced by reduced protein corona effects. Broad‐spectrum inhibition with Batimastat (BB‐94) suppressed activity in all samples except ascites TFF‐UF, suggesting that additional, non‐metalloproteases were co‐purified. Selective inhibition with GI254023X demonstrated a significant contribution of ADAM10, consistent with earlier studies on its EV association and substrate specificity [24]. Western blotting confirmed ADAM10 detection in all preparations, with the active membrane‐bound form particularly abundant in ES‐2 EVs.

These findings underscore the importance of ADAM10 not only as a mechanistic EV marker but also as a potential functional biomarker for clinical translation. Although there seems to be an equilibrium between soluble and membrane‐bound ADAM10, we likely detect both activities, assuming even the cleaved ADAM10 remains bound to purified EVs.

In agreement with current reports, UC‐DG and TFF‐SEC yielded the highest enrichment of canonical EV proteins with the lowest levels of contaminants, whereas TFF‐UF showed increased proportions of lipoproteins and secreted proteins, and UC‐UC displayed higher enrichment of intracellular components.

Several methodological constraints should be considered when interpreting our results. Neither the 0.22‐µm filtration step nor the TFF procedure were performed under controlled shear‐rate or pressure conditions, which may affect EV membrane integrity and the retention of loosely bound corona proteins. Filtration‐ and flow‐induced shear stress has been shown to damage proteins and viruses and may similarly impact EV‐associated structures and functions [26]. Moreover, our TFF workflow did not include a diafiltration step; ultrafiltration alone does not achieve complete buffer exchange or efficient depletion of low‐molecular‐weight or soluble proteins, particularly in protein‐rich matrices such as ascites. These matrix‐dependent effects likely contribute to the pronounced differences observed between ascites‐ and ES‐2‐derived TFF‐UF preparations, including increased non‐vesicular background material. Finally, as a size‐based separation principle, TFF is inherently prone to co‐isolation of lipoproteins overlapping with EVs in size, whereas density‐based approaches such as UC‐DG provide higher stringency at the expense of yield and functional activity. Collectively, these considerations highlight that isolation principles intrinsically shape EV composition and functional readouts, underscoring the need to interpret results within the context of method‐specific constraints.

The observed difference between ascites‐ and ES‐2‐derived EVs in the TFF‐UF workflow is likely to be matrix‐dependent, with the abundance of soluble proteins and lipoproteins present in ascites, which are retained during ultrafiltration, resulting in a UF retentate dominated by these components. In contrast, the ES‐2 milieu is protein‐free, resulting in a comparatively purer retentate. This corresponds to earlier studies demonstrating that density gradients and SEC most effectively deplete lipoprotein co‐isolates, while ultrafiltration and concentration steps are prone to protein carry‐over [4, 23, 25]. While TFF‐UF maximizes recovery, TFF‐SEC balances yield and purity, UC‐UC provides moderate recovery with reduced protein load, and UC‐DG achieves the highest stringency at the expense of yield. Known and putative ovarian cancer biomarkers were detectable across nearly all preparations, although their presence does not necessarily imply strict EV association, underlining the need for careful interpretation of biomarker recovery in translational context. Our systematic, dual‐source comparison across four methods, combined with orthogonal sizing and morphology validation, therefore extends current reports by providing a decision‐making framework for selecting isolation strategies tailored to scientific or translational goals in EV research.

Thus, our data demonstrate that, even under particle‐normalized conditions, the choice of isolation method substantially shapes the detectable EV proteome and EV‐function. Clear clustering of workflows in PCA and variable overlaps in marker sets extend the recent MISEV2023 guidelines, which emphasize that separation principles (density‐, size‐, or flow‐based) define both the yield and the interpretability of EV‐associated components and co‐isolates [22, 27]. At the same time, our results underscore the inherent limitations of relying solely on guideline‐based marker panels. The recovery of canonical MISEV proteins was highly dependent on the isolation strategy, and categories intended to differentiate vesicular from non‐vesicular components were variably retained or lost across workflows. This variability illustrates that marker‐based evaluation alone cannot fully capture isolation performance, and may even mislead when applied without considering translational suitability of EV preparations. Consequently, guideline checklists should be viewed as a framework rather than a definitive standard, with careful contextual interpretation required to ensure meaningful cross‐study comparisons.

In addition, we suggest that functional, cell‐free assays such as the FRET‐based protease readout employed provide a robust complement to structural and compositional profiling, as they circumvent the interpretational pitfalls of cell‐based assays [4, 25]. Incorporating such functional assays into isolation benchmarking pipelines will be critical for guiding the development of clinical applications.

## Author Contributions

**Conceptualization**: Elke Pogge von Strandmann, Christian Preußer, Jörg W. Bartsch; **Investigation**: Dolores J. Salander, Christian Preußer, Jörg W. Bartsch, Yukai Wang, Witold Szymanski; **Resources**: Elke Pogge von Strandmann, Silke Reinartz, Johannes Graumann, Jörg W. Bartsch; **Formal Analysis**: Christian Preußer, Dolores J. Salander, Witold Szymanski, Yukai Wang, Johannes Graumann, María Gómez‐Serrano; **Writing – Original Draft**: Christian Preußer, Elke Pogge von Strandmann; **Writing – Review and Editing**: Christian Preußer, Elke Pogge von Strandmann, Johannes Graumann, Jörg W. Bartsch, Dolores J. Salander, Yukai Wang.

## Conflicts of Interest

The authors declare no conflicts of interest.

## Supporting information




**Supporting File 1**: adhm70774‐sup‐0001‐SuppMat.docx.

**Supporting File 2**: adhm70774‐sup‐0002‐TablesS1‐S2.xlsx.

## Data Availability

The data that support the findings of this study are available from the corresponding author upon reasonable request.
