# Supplementary material for: Isolation Defines Identity: Functional Consequences of Extracellular Vesicle Purification Strategies
Source: Adv Healthc Mater. 2026 Jan 19;15(13):e04684. doi: 10.1002/adhm.202504684 (PMC13058776; doi:10.1002/adhm.202504684)
Supplement: Supplementary file 1 — Supporting File 1: adhm70774‐sup‐0001‐SuppMat.docx. [file ADHM-15-0-s001.docx]

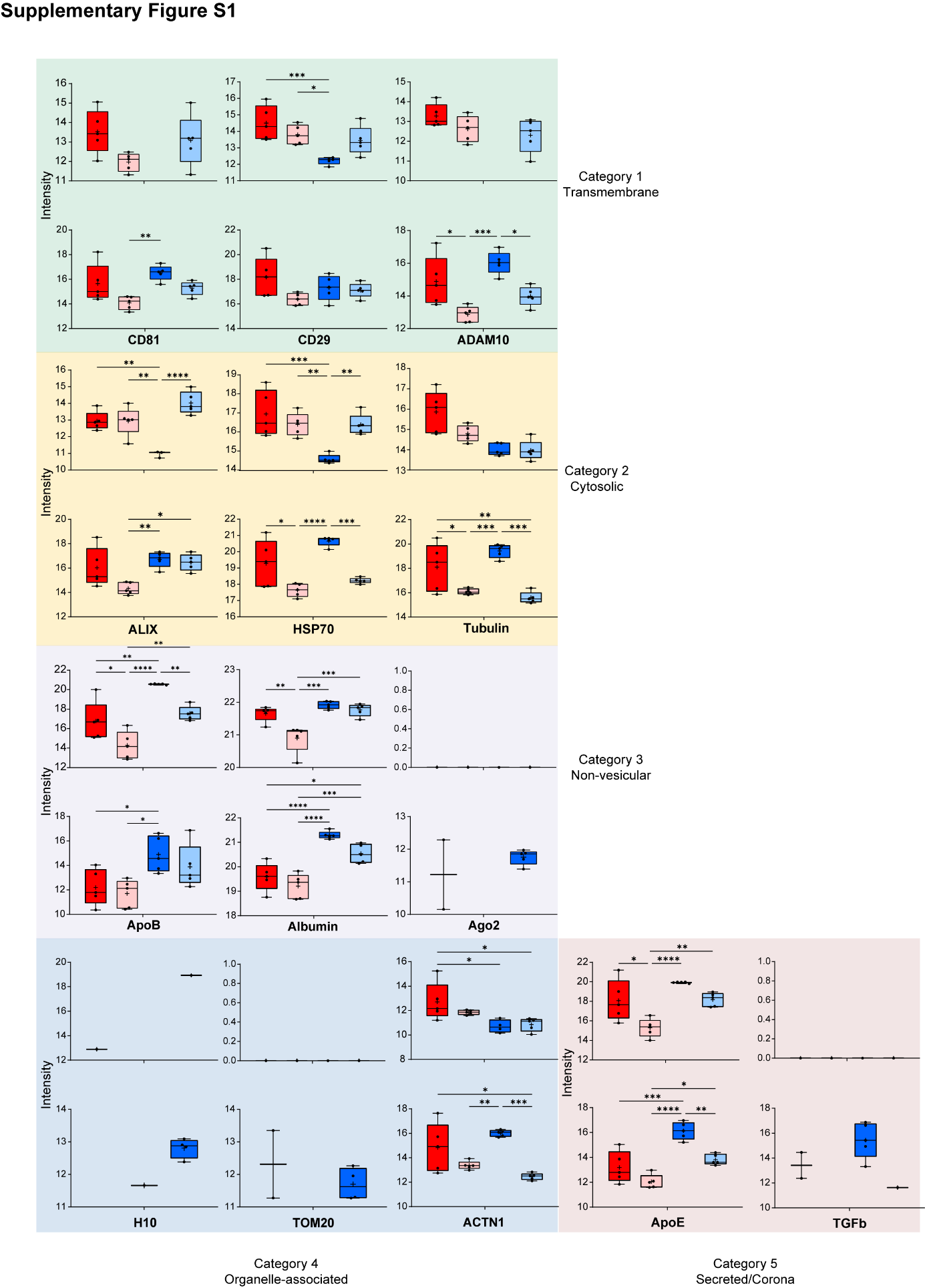


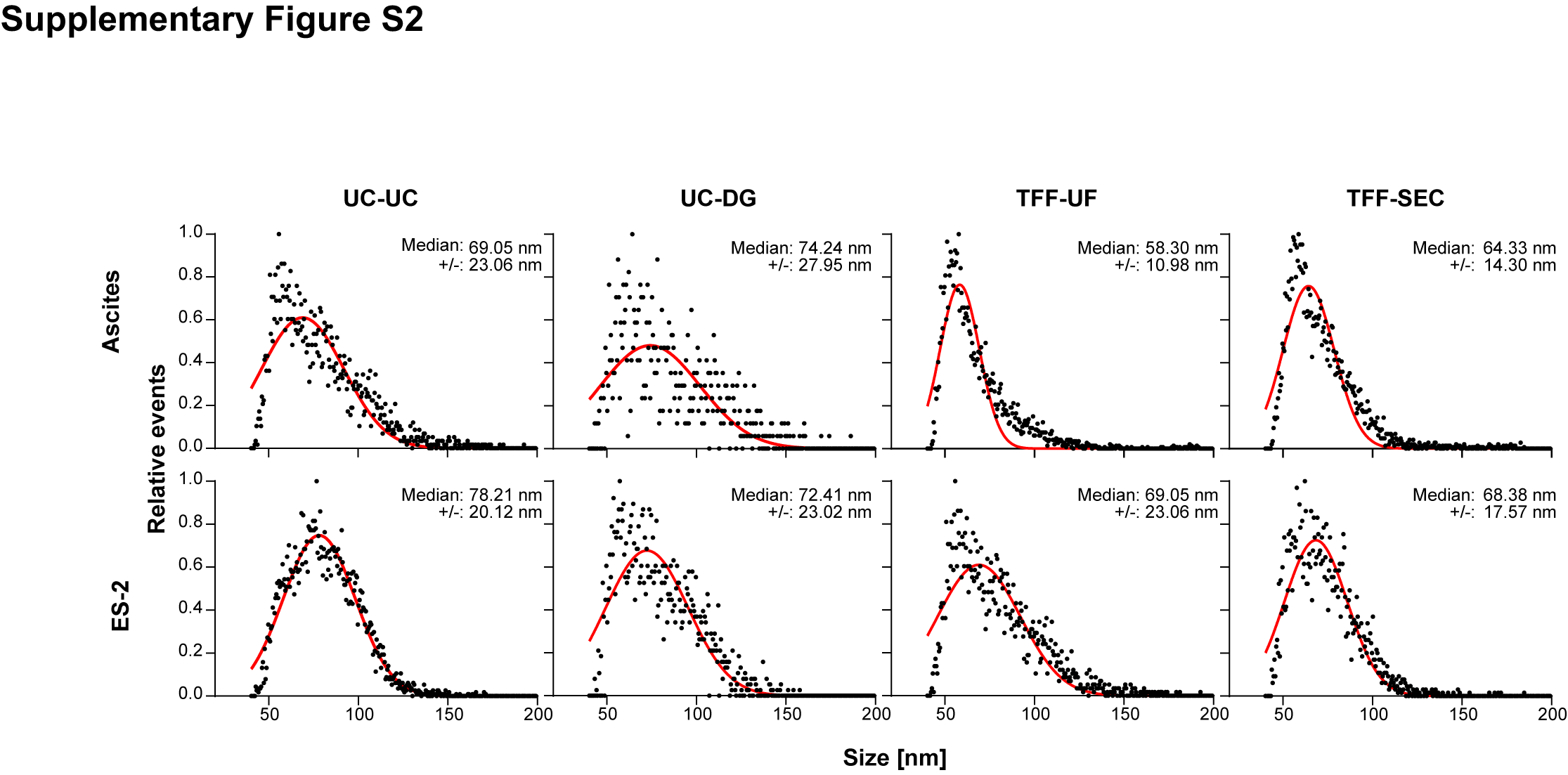


**Supplemantary Figure S3**

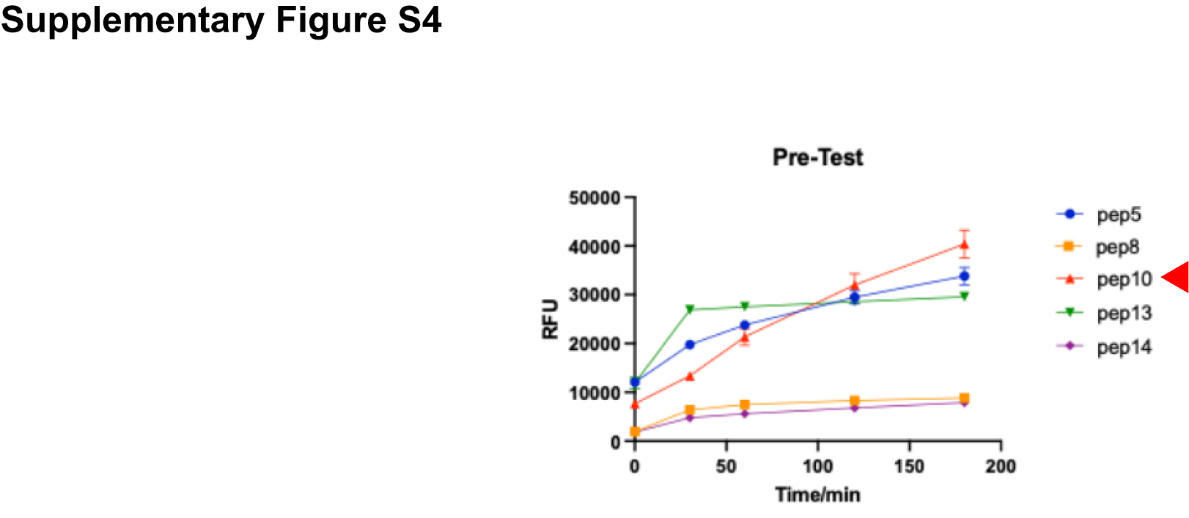


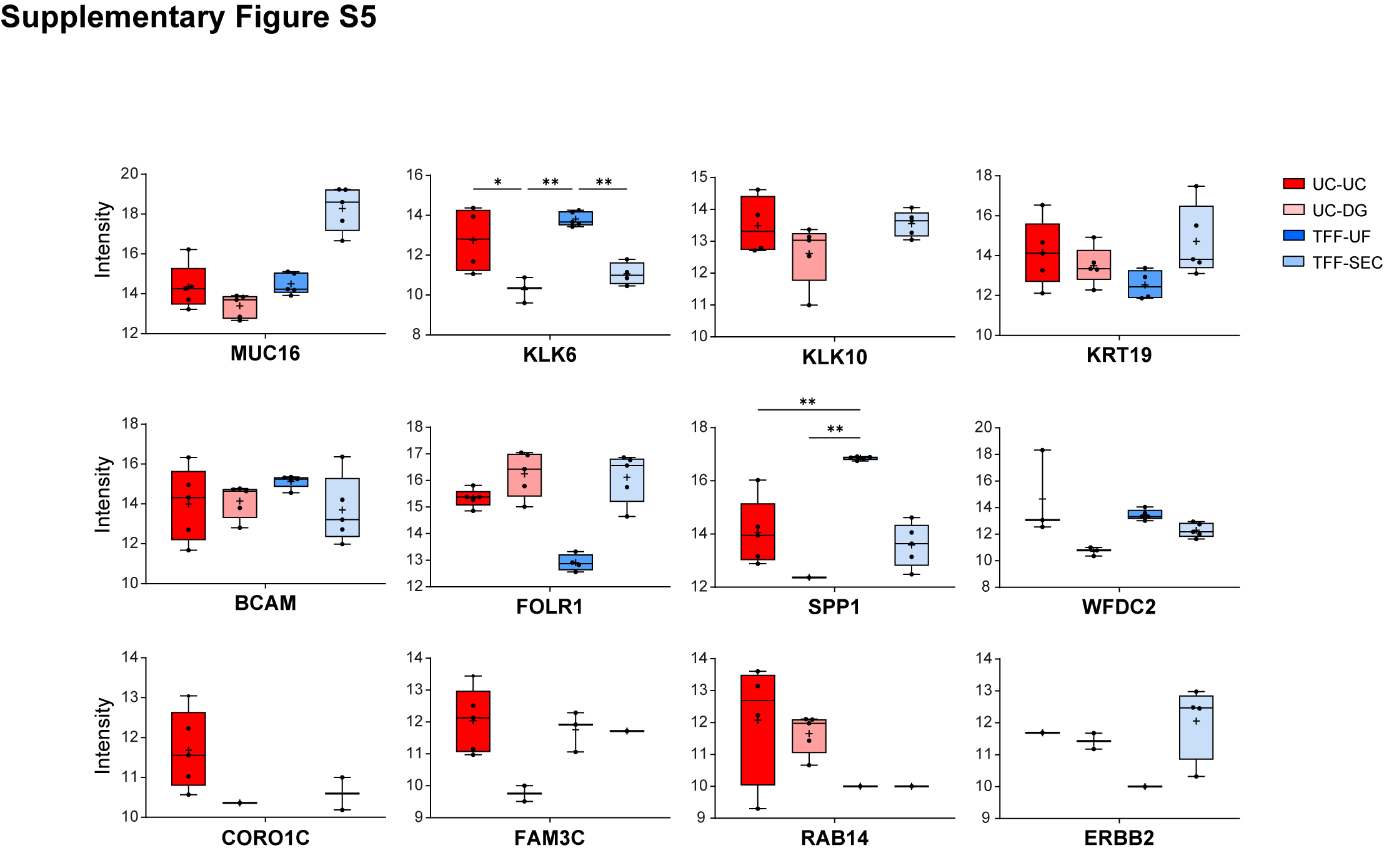


**Supplementary Figure S1. Detection of representative proteins from EV and non-EV categories across isolation methods.**

Boxplots (mean ± SEM) display MaxLFQ intensities of selected proteins representing different categories: (1) transmembrane (CD81, CD29, ADAM10), (2) cytosolic (ALIX, HSP70, Tubulin, Ago2, ACTN1), (3) non-vesicular contaminants (ApoB, Albumin), (4) organelle-associated (TOM20), and (5) secreted/corona proteins (ApoE, TGFβ). Data are shown for UC-UC (dark red), UC-DG (light red), TFF-UF (dark blue), and TFF-SEC (light blue). Asterisks indicate significant differences between methods (*p < 0.05, **p < 0.01, ***p < 0.001, ****p < 0.0001; two-way ANOVA with Tukey’s multiple comparisons).

**Supplementary Figure S2. Size distribution of EVs from ascites and ES-2 preparations measured by nano-flow cytometry.**

Relative event counts are plotted against vesicle diameter (nm) for each isolation method (UC-UC, UC-DG, TFF-UF, TFF-SEC). Median particle diameters ± spread are indicated for each condition.

**Supplementary Figure S3. Single-particle phenotyping of EVs by nano-flow cytometry.**
EVs isolated from ascites and ES-2 cell culture supernatants were fluorescently labeled with FITC-conjugated antibodies to CD9, CD81, and CD29, or with PE-conjugated CD63. Bivariate dot plots of fluorescence versus side scatter (SSC) are shown for each isolation method (UC-UC, UC-DG, TFF-UF, TFF-SEC). Double-positive events (e.g., CD9/CD63) are depicted on the right-hand side.

**Supplementary Figure S4. Screening of quenched peptide substrates for EV-associated protease activity.**

Five different quenched synthetic peptide substrates (pepDAB5, pepDAB8, pepDAB10, pepDAB13, and pepDAB14, see Miller et al., 2011) were tested for their ability to detect enzymatic activity in EVs. Note that only pepDABs 5 and 10 provided a linear response, with pepDAB10 being more selective for ADAM10 compared to pepDAB5.

**Supplementary Figure S5.** Expression profiles of established and putative ovarian cancer biomarkers across EV isolation methods. Boxplots display relative MaxLFQ intensities of representative proteins including MUC16, KLK6, KLK10, KRT19, BCAM, FOLR1, SPP1, WFDC2, CORO1C, FAM3C, RAB14, and FOLR1. Asterisks indicate significance (*p < 0.05; **p < 0.01; two-way ANOVA with Tukey’s multiple comparisons)
